# Molecular Dynamics Simulations of A27S and K120A Mutated PTP1B Reveals Selective Binding of the Bidentate Inhibitor

**DOI:** 10.1155/2019/9852897

**Published:** 2019-01-08

**Authors:** Xi Chen, Xia Liu, Qiang Gan, Changgen Feng, Qian Zhang

**Affiliations:** ^1^State Key Laboratory of Explosion Science and Technology, Beijing Institute of Technology, No. 5, Zhongguancun South Street, Haidian District, Beijing 100081, China; ^2^College of Science, China Agricultural University, Beijing 100193, China

## Abstract

Protein tyrosine phosphatase 1B (PTP1B) is considered a potential target for the treatment of type II diabetes and obesity due to its critical negative role in the insulin signaling pathway. However, improving the selectivity of PTP1B inhibitors over the most closely related T-cell protein tyrosine phosphatase (TCPTP) remains a major challenge for inhibitor development. Lys120 at the active site and Ser27 at the second pTyr binding site are distinct in PTP1B and TCPTP, which may bring differences in binding affinity. To explore the determinant of selective binding of inhibitor, molecular dynamics simulations with binding free energy calculations were performed on K120A and A27S mutated PTP1B, and the internal changes induced by mutations were investigated. Results reveal that the presence of Lys120 induces a conformational change in the WPD-loop and YRD-motif and has a certain effect on the selective binding at the active site. Ser27 weakens the stability of the inhibitor at the second pTyr binding site by altering the orientation of the Arg24 and Arg254 side chains via hydrogen bonds. Further comparison of alanine scanning demonstrates that the reduction in the energy contribution of Arg254 caused by A27S mutation leads to a different inhibitory activity. These observations provide novel insights into the selective binding mechanism of PTP1B inhibitors to TCPTP.

## 1. Introduction

Protein tyrosine phosphatases (PTPs) are a superfamily of enzymes involved in controlling a variety of cellular response, including cellular growth, differentiation, metabolism, and immunity [1]. Protein tyrosine phosphatase 1B (PTP1B), the most representative member of this superfamily, is identified as a promising target in type II diabetes and obesity [2]. It was the first PTPase to be cloned and purified from human placenta by Tonks et al. [3]. The landmark study of PTP1B knockout mice showed that loss of PTP1B led to an increase in insulin sensitivity and maintenance of blood glucose level in a high-fat diet [4]. Further studies confirmed that PTP1B dephosphorylates both the insulin receptor and the leptin receptor substrate-1 [5, 6]. These results confirm the critical negative regulation of PTP1B in insulin and leptin signaling. In addition, PTP1B is also observed to be overexpressed in breast tumor, which highlights a new treatment for breast cancer [7].

Although a variety of PTP1B inhibitors have been successfully discovered, such as pTyr mimetics and natural products [8], the highly conserved catalytic domain of PTP1B makes it difficult to increase the inhibitor selectivity over other PTPs. In particular, T-cell protein tyrosine phosphatase (TCPTP) is the closest cousin of PTP1B, which shares a high sequence homology with PTP1B in the catalytic domain (74% identity) [9]. TCPTP knockout mice died in 3-5 weeks after birth due to reduced bone marrow cells and impaired T-cell and B-cell function, suggesting that TCPTP participate in the regulation of hematopoiesis and the immune system [10]. The study of PTP1B and TCPTP cross-deficient mice demonstrates that the functions of these two enzymes are not redundant in IFN-*γ* signaling [11]. As the two enzymes are distinct in physiological function, it is necessary for PTP1B inhibitors to have sufficient selectivity over TCPTP.

**Figure 1:**
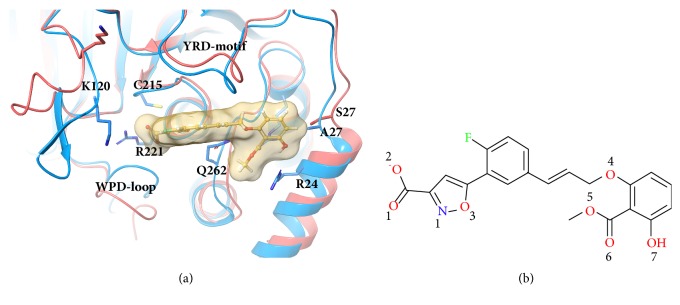
(a) Superimposed structures of PTP1B (PDB ID: 1Q1M) and TCPTP (PDB ID: 1L8K) which are shown in blue and red, respectively. The inhibitor is shown by ball-and-stick with a transparent surface. (b) Structure of the inhibitor labeled with oxygen and nitrogen atoms.

The key factor underlying the binding selectivity of PTP1B inhibitor is still debated. One promising strategy for increasing the selectivity is to target both the active site and the adjacent second pTyr binding site (Figure 1(a)). Puius et al. [12] first discovered the second pTyr binding site when they analyzed the crystal structure of PTP1B complexed with bis-(para-phosphopheny) methane. Arg24, Arg254, and Gln262 at this shallow pocket are identified as favorable residues to generate interactions with inhibitors. Although some of the inhibitors in the subsequent studies have succeeded in achieving good selectivity, which was discussed in several reviews [8, 13], further selective optimization targeting this site was not ideal as these residues are highly conserved [14, 15]. Fortunately, the adjacent differential residues bring more possibilities. Ala27/Ser29 (PTP1B/TCPTP) at the second position showed its potential as the selectivity of the inhibitor increased to 7.2-fold when interacting with this residue [16]. Our previous research has found that this difference increases selectivity by affecting the interactions of inhibitors with Arg24 [17]. Besides, Lys120/Lys122 is also considered by some researchers [18–20]. Our previous research also found that the R-loop differs in orientation between PTP1B and TCPTP, as it participates in the binding of inhibitors at the active site in PTP1B, but absent in TCPTP [17], and this conformational difference may affect the binding of PTP1B at the active site. However, the dynamic behavioral differences between Ala27/Ser29 and Lys120/Lys122 are not clear, which undoubtedly limits the development of inhibitors targeting this site.

In this paper, we aimed to investigate the difference internal behaviors of Ala27/Ser29 and Lys120/Lys122 in selective binding of inhibitors. The internal behaviors of PTP1B-inhibitor complex and mutants at A27S and K120A were investigated by molecular dynamics simulations. The most representative bidentate inhibitor (Figure 1(b)), with the best selectivity (23.77-fold) in all crystal structures of PTP1B complexes [21], was used as a probe to detect the effect of mutations. It is a bidentate inhibitor that binds to both the active site and the second pTyr binding site. The conformational changes and energy differences were analyzed to further explore the key factors affecting binding selectivity.

## 2. Methods

### 2.1. System Preparations

The initial structure of PTP1B was retrieved from the Protein Data Bank (PDB code: 1Q1M) and was then submitted to generating K120A and A27S mutations by UCSF Chimera 1.10.1 software [22]. Receptors were prepared and missing atoms of the terminal residues were fixed by the tLEaP module in Amber 14 [23] and the protonation states were set to pH 7.4 by PROPKA 3.0 [24]. The RESP partial charges of inhibitor were calculated by the Amber antechamber program [25], based on the electrostatic potentials calculated by Gaussian 03 at the (HF)/6-31G*∗* level [26]. Each system was solvated by a cubic water box using TIP3P water molecules with a side length of 10 Å, and the net charge was neutralized by sodium ions with ff99SB [27] force field.

### 2.2. Molecular Dynamic Simulations

The MD simulations were performed using Amber 14 package, with the force field of Amber ff99SB [27] and general Amber force field (GAFF) [28] for proteins and inhibitor, respectively. We used a protocol similar to our previous study [29]. The systems were first minimized by (1) the 1000 steps of steepest descent and the 1000 steps of the conjugate gradient, under a harmonic constraint of 10.0kcal/(mol·Å^2^) on heavy atoms; (2) relaxing the entire system by 5000 steps of steepest descent and 15 000 steps of the conjugate gradient. Then, the system was gradually heated to 300 K by a 50 ps NVT simulation and was equilibrated by a 500 ps NPT simulation at 1 atm. The temperature and pressure were kept by the Langevin thermostat and the Berendsen barostat with a relaxation time of 2 ps, respectively. Finally, each system was subjected to a 50 ns NPT simulation without restraint. During simulations, the SHAKE algorithm [30] was applied to all hydrogen atoms with a time step of 2 fs.

The root-mean-square deviation (RMSD), the root-mean-square fluctuation (RMSF) values, and hydrogen bonds (H-bonds) were calculated by the ptraj module in Amber [31]. RMSF during the equilibrium was calculated with the reference of the time average structure. Clustering analysis using the k-means clustering algorithm was also performed on the equilibrium trajectories by the ptraj module based on RMSD of heavy atoms. The initial frames were selected randomly and the sieve size was set to 10 for the total 5000 frames of trajectories. The residue-residue cross-correlations were calculated by the Bio3D package [32] to determine how mutations affect the internal dynamics of protein conformations. The cross-correlation coefficient of C*α* atoms was calculated according to the average structure. The residue interaction networks (RINs) of average structures were analyzed using the Ring web server [33], and the RINs graphs were generated by Cytoscape software [34]. Based on the RINs, important residues were identified and distances between the residue pairs were monitored.

### 2.3. Binding Free Energy Calculations and Alanine Scanning

The molecular mechanics Poisson−Boltzmann surface area (MM-PBSA) method [35] was employed. To increase the precision of MM-PBSA calculation, ten independent NPT runs of 250 ps after 50 ns of production were performed for each system, and the average binding free energies were calculated. This method has been confirmed to be effective by Genheden S et al. [36]. The binding free energy Δ*G*_bind_ was evaluated by

(1) ΔGbind=Gcom−Grec+GligΔGbind=ΔEinternal+ΔEele+ΔEvdw+ΔGPB+ΔGSA–ΔTS

Δ*G*_bind_ is contributed by the internal energy (Δ*E*_internal_), the electrostatic energy (Δ*E*_ele_), van der Waals energy (Δ*E*_vdW_), the electrostatic solvation energy (Δ*G*_PB_), the nonpolar contributions (Δ*G*_SA_), and the entropy. The solute dielectric constant of 1 and the exterior dielectric constant of 80 was applied. The Δ*G*_PB_ term was calculated by using the PB solver in MMPBSA.py module in Amber 14. The nonpolar contribution (Δ*G*_SA_) was evaluated by Δ*G*_SA_ = 0.03780 ×Δ*SASA* − 0.5692 + *E*_dispersion_, where SASA was the solvent accessible surface area and *E*_dispersion_ was the dispersion term [37]. The molecular surface was determined by sphere probes with a radius of 1.4 Å. The* nmode* program in Amber was applied to calculate the entropy term. Alanine scanning was performed to determine the energetic contribution of each residue, and the energy difference before and after the mutation was calculated by ΔΔ*G*= Δ*G*_wild_ – Δ*G*_mutant_.

## 3. Results and Discussion

In this article, we interested in the different roles of Ala27/Ser29 and Lys120/Lys122 in the selective binding of a bidentate inhibitor to the active site and the second pTyr binding site. We focus on how they affect the conformation and interaction networks of the surrounding region, especially WPD-loop, YRD-motif, and loop28-32, which in turn affect binding of the inhibitor. The highly conserved WPD-loop prefers to be in a closed conformation binding with substrates, and Asp181 in this loop act as an essential general acid in the catalyzed reaction [38]. The inhibitors used herein are designed for the closed state of PTP1B, achieving its bidentate binding by forming hydrogen bonds with Arg221, Arg24, Arg254, and Gln262 [21]. Since the WPD-loop of the only crystal structure of TCPTP (1L8K) is in an unfavorable “open” state [9], which requires long-timescale simulations to be in the "closed" state, mutations of A27S and K120A on PTP1B were carried out.

### 3.1. Stability and Overall Structural Flexibility of WT, A27S, and K120A Systems

**Figure 2:**
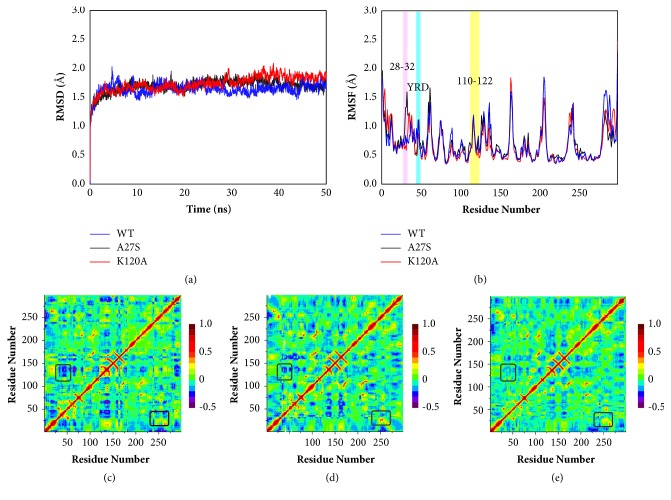
**Overall conformational changes of WT, A27S and K120A systems in MD simulation.** (a) The RMSD profiles for the backbone atoms of the WT, A27S, and K120A system. (b) The RMSD profiles for the side-chain atoms of the WT, A27S, and K120A system obtained from 50 ns MD simulations. The residues 28-32, YRD-motif, and the loop 110-122 were labeled. Dynamic cross-correlation matrices of residue fluctuation from the equilibrated simulations of the (c) WT, (d) A27S, and (e) K120A system.

The preliminary calculation of RMSD for the C*α* atoms was performed with reference to the initial structure, and the result suggests that the WT and mutant systems reach equilibrium after 10 ns (Figure 2(a)). RMSF was then computed to evaluate the flexibility of each residue. As shown in Figure 2(b), the most obvious difference in fluctuation appears in residues 28-32. The RMSF value of this region in the A27S system is much higher than in the other two systems, indicating that the mutation A27S affects the flexibility of residues 28-32. The fluctuations of loop 110-122 and the WPD-loop present a high degree of similarity in all systems, suggesting that the K120A mutation does not bring specific conformational change for these two loop structures. It is worth noting that mutation K120A obviously reduces the RMSF of YRD-motif (46-48), which suggests that the flexibility of YRD-motif is affected by Lys120. YRD-motif is a charged region closed to the active site as labeled in Figure 1. Together with WPD-loop, it provides *π*-stacking interactions for the inhibitor [39].

To understand the internal dynamic changes caused by mutations, cross-correlation analysis was performed on equilibrium trajectories. Figures 2(c)–2(e) show the results of the three systems, where the high correlated region in red indicates the motion in the same direction and the high anti-correlation region in blue indicates the motion in the opposite direction. Notably, a stronger positive correlation between Arg254 and the loop 28-32 was observed in the A27S system. By analyzing the correlation coefficient of Arg254 to each residue (Figure S1), this correlated motion is confirmed to be enhanced by the mutation A27S. The interaction between Arg254 and Ser27 may lead to conformational changes in Arg254, which in turn affects the binding at the second pTyr binding site. This interaction also affects the conformation of the loop in which Ser27 is located, which explains the greater flexibility of loop28-32 in the A27S system. Additionally, the anticorrelated motion of loop110-122 with YRD-motif was found to be decreased by the mutation K120A, which cause differences in the conformation of YRD-motif.

**Figure 3:**
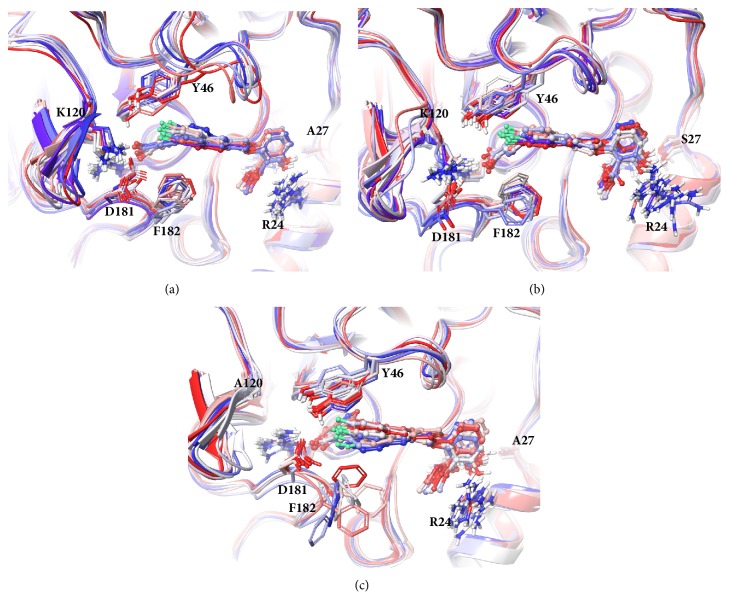
**Average structures of the top 10 clusters of** (a)** WT,** (b)** A27S, and** (c)** K120A systems.** The structure is colored from red to blue, representing the cluster from large to small. The inhibitors are represented in the ball-and-stick model.

Clustering analysis on the equilibrium trajectories was performed to characterize the conformational changes in different systems. The number of clusters was set according to the values of Davies-Bouldin index (DBI) and the pseudo F-statistic (pSF) (Figure S2). The top 10 clusters cover over 70% of conformations (Table S1) and the average structures of each cluster were extracted and superimposed as shown in Figure 3. At the active site, the binding positions of the inhibitor at the active site are relatively concentrated in the WT and A27S systems. Interestingly, the orientations of the side chain of Lys120 in these two systems do not converge in a single direction, but in different directions around the region between WPD-loop and YRD-motif. Besides, under the influence of Lys120, the conformations of YRD-motif of different clusters in WT and A27S systems are less concentrated than that of K120A system. In the K120A system, the positions of inhibitor at the active site are more dispersed, which are closer to the WPD-loop. Phe182 in the K120A system has distinct orientations in different clusters, which results in the inhibitor binding at the active site being inferior to other systems. At the second pTyr binding site, there is a significant difference in the orientation of Arg24 in the A27S system. In addition, the conformations of loop28-32 are more dispersed than the other two systems (Figure S3), which also reflects that its flexibility is greater. However, further analysis of the interaction network and energy calculations is needed to determine whether the interactions between Lys120, YRD-motif, and WPD-loop will affect the binding affinity at the active site and what role Ser27 plays in the second pTyr binding site.

### 3.2. Residue Interactions at the Active Site

**Figure 4:**
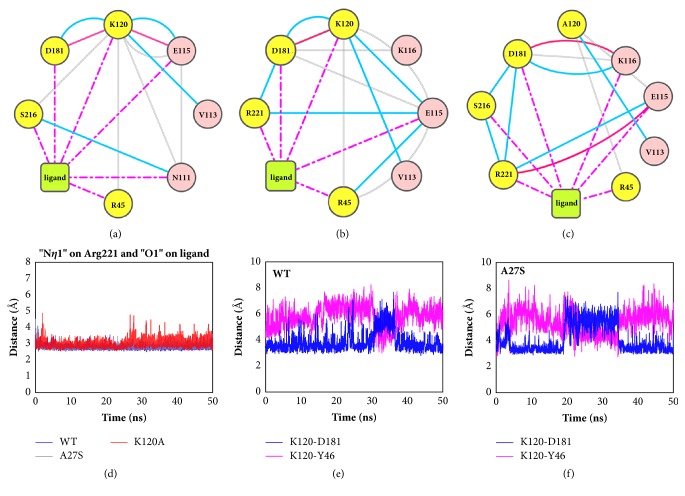
**Residue interactions and conformational changes at the active site.** Residue interaction networks for the (a) WT, (b) A27S, and (c) K120A systems showing the interactions at the active site. The blue line represents the H-bond, the gray line represents the van der Waals interaction, the pink solid line represents the salt bridge, and the magenta dotted line represents the generic contact with their closest atom. The distances between (d) the N*η*1 atom of Arg221 and the O1 atom of the ligand, and the N*ζ* atom of Lys120 and the C*γ* atom of Asp181 for the (e) WT and (f) A27S systems.

Then, we focused on the residue interactions at the active site. In order to explore the internal changes caused by mutations, the residue interaction networks of average structures were analyzed. As shown in Figures 4(a)–4(c), Lys120 in both WT and A27S systems participate in the binding of inhibitors by salt bridges. The van der Waals interactions between Lys120/Ala120 and Arg45 were observed in all systems. Considering the larger steric hindrance of Lys120 than Ala, the conformation of YRD-motif is more susceptible to Lys120, which may be responsible for the anticorrelation between YRD-motif and Lys120 in the DCCM analysis. This conformational difference also leads to the fact that Tyr46 and the aromatic rings of the inhibitor in the K120A system are closer to the P-loop than other two systems, as shown in Figure S4. Asp181 at the WPD-loop is observed to form H-bonds with Lys120. Under the binding of a substrate, WPD-loop usually shifts from an open conformation to a closed state [38]. The attractions of Lys120 promote the approach of Asp181 to the active site, thereby strengthening the closure of the WPD-loop. However, we subsequently found that this attraction to Asp181 in the K120A system can be compensated by the salt bridge with Lys116, which explains why the conformational difference of WPD-loop induced by Lys120 is not as large as expected.

In all three systems, similar binding patterns of the inhibitor at the active site are observed by forming H-bonds with Cys215, Gly220, and Arg221 (Figure S5). The distances between these residues at the active site were monitored. As shown in Figure S6A, the distances between Cys215 and O2 atom of the inhibitor were stable at about 3 Å in all of the three systems, which proves that the inhibitor binds firmly to the active site. However, the fluctuation of the distances between Arg221 and the O1 atom of the inhibitor in K120A system is larger than that in WT and A27S systems (Figure 4(d)). This result provides evidence that the absence of Lys120 affects the binding stability of the inhibitor to the active site. By comparing the changes in distances of Lys120-Asp181 and Lys120-Tyr46 in the WT and A27S systems (Figures 4(e)-4(f)), it is clear that Lys120 swings between WPD-loop and YRD-motif. The steric hindrance between Lys120 and YRD-motif caused the main chain of YRD-motif to move about 1 Å from the active site (Figure S6B), providing more space for Tyr46 to adjust the position. This result is consistent with the analysis of RMSF. Lys120 is found to stabilize the orientation of the side chain of Tyr46 in YRD-motif, as the fluctuation of distance between side chains of Tyr46 and Arg221 in the WT and A27S system were smaller than in K120A system (Figure S6C). Considering the hydrogen bond with Asp181 and van der Waals interaction with Arg45, Lys120 acts as an agent between them through WPD-loop and YRD-motif. This effect of Lys120 is beneficial to the binding of the inhibitor at the active site, due to the *π*-stacking interaction between Tyr46, Phe182, and the central phenyl ring of the inhibitor. The absence of Lys120 results in a relatively unstable binding site of the inhibitor at the active site, further making the orientation of Phe182 differ significantly in clustering analysis of K120A system. Since the orientation of Lys122 in TCPTP is away from the active site by forming a hydrogen bond with Glu117 [17], it will have less effect on binding of inhibitor at the active site than that in PTP1B.

### 3.3. Residue Interactions at the Second pTyr Binding Site

**Figure 5:**
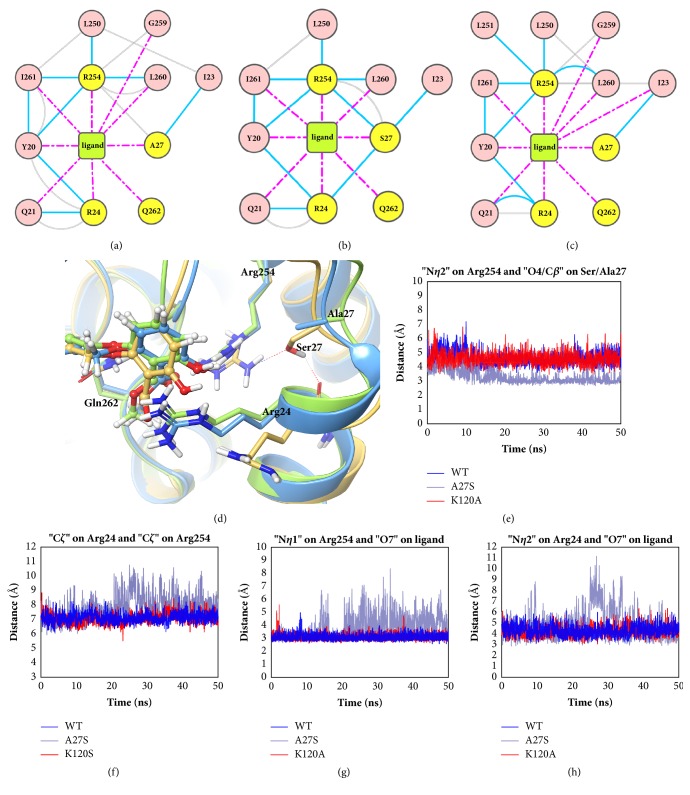
**Residue interactions and conformational changes at the second pTyr binding site.** Residue interaction networks for the (a) WT, (b) A27S, and (c) K120A systems showing the interactions at the second pTyr binding site. The blue line represents the H-bond, the gray line represents the van der Waals interaction, the pink solid line represents the salt bridge, and the magenta dotted line represents the generic contact with their closest atom. (d) Average structures of the WT, A27S, and K120A systems superimposed at the second pTyr binding site, shown in green, yellow, and blue, respectively. The distances between (e) the N*η*2 atom of Arg254 and the O4/C*β* atom of the Ser27/Ala27, (f) the C*ζ* atom of Arg24 and the C*ζ* atom of Arg254, (g) the N*η*1 atom of Arg254 and the O7 atom of the ligand, and (h) the N*η*2 atom of Arg24 and the O7 atom of the ligand.

The ligand interaction diagram (Figure S5) indicates that H-bonds with Arg254 and Gln262 are essential for the binding of the inhibitor at the second pTyr binding site. To gain a more detailed insight, the residue interaction networks at the second pTyr binding site were also explored (Figures 5(a)–5(c)).

For the WT and K120A systems, the residue networks are quite similar. Arg254 in both systems form H-bonds with Leu260 and Ile261 on the Q-loop, thereby stabilizing the orientation of its side chain. Compared to Arg254, Arg24 is more flexible as it mainly interacts with surrounding residues through the backbone, while its side chain is unconstrained. In the A27S system, Ser27 was found to form additional H-bonds with both Arg254 and Arg24, which leads to a conformational difference at the second pTyr binding site. Ser27 in the A27S system was observed to get 1.5 Å closer to Arg254 in A27S system after 20 ns of simulations (Figure 5(d)). This result reveals the reason why the flexibility of loop 28-32 is different from the other two systems in the RMSF analysis. Under the influence of the conformational change of loop 28-32, Arg24 shifts away from the second pTyr binding site, as the fluctuation range of the distance Arg24-Arg254 in the A27S system is 3 Å larger than in other systems (Figure 5(f)). The conformational variation of Arg24 and Arg254 further affects the binding of the inhibitor at the second pTyr binding site. The distance between the O7 atom of the inhibitor in the A27S system and the N*η*1 atom of Arg254 is not maintained at 3 Å as in the cases of WT and K120A but fluctuates between 3 and 8 Å (Figure 5(g)). Similar results also appear in the distance between the O6 atom of the inhibitor and the N*η*2 atom of Arg254 (Figure S6D). The above results prove that the methyl salicylate of the inhibitor in the A27S system does not form stable H-bonds with Arg254 as in the WT and K120 systems, but gradually moves away from Arg254. It is worth noting that under the H-bond between Gln262 and the O4 atom of the inhibitor (Figure S6E), the methyl salicylate group cannot completely leave the second pTyr binding site. Besides, Arg24 in the A27S system, despite the conformational change, still hinders the departure of the methyl salicylate group, as the O7 atom of the inhibitor gradually approaches Arg24 to 4 Å after being away from Arg254 (Figure 5(h)).

### 3.4. Binding Free Energy Calculations and Hydrogen Bond Analysis

The binding free energies for the WT, A27S, and K120A systems were calculated using the MM-PBSA method. As shown in Table 1, the result of the WT system is -8.03 ± 0.3 kcal/mol, consistent with the experimental value of −7.33 kcal/mol [21]. The result of the K120A system is -6.84 ± 0.34 kcal/mol, which is slightly smaller than that of the WT system, indicating that Lys120 brings a difference in binding affinity. In particular, the binding free energy for the A27S system is −4.86 ± 0.33 kcal/mol, which is the lowest among the three and is similar to the experimental value for the inhibitor-TCPTP complex [21]. It is clear that Ser27 has a more significant effect on inhibitor selectivity than Lys120. Individual energetic terms show that electrostatic and van der Waals interactions are the main driving forces of the binding process, whereas polar solvation term presents unfavorable effect. The K120A system has the lowest Δ*E*_*ele*_ due to the lack of electrostatic interaction between Lys120 and the inhibitor. However, the unfavorable solvation term Δ*G*_*solv*_ caused by Lys120 is also less compensated than other systems.

**Figure 6:**
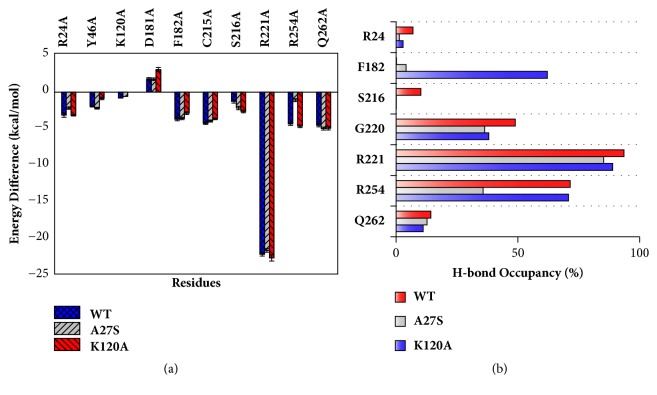
(a) Differences in binding free energies of important residues calculated by the MM-PBSA alanine scanning method. (b) Comparison of hydrogen bond occupancy of important residues during MD simulations.

Alanine scanning mutagenesis and H-bond analysis were applied to further verify key residues responsible for the selectivity of the bidentate inhibitor. Results are depicted in Figures 6(a) and 6(b) and detailed information is shown in Tables S2-S3.

In all systems, Arg221 exhibits the largest energy difference among all residues, and the H-bond occupancy is above 85%, which illustrates that Arg221 is the dominant contributor to binding at the active site. Besides, H-bonds between Gly220 and the isoxazole group of inhibitor are observed in each system, with the occupancy of more than 38%. Obvious differences were found in A27S system terms of energy contributions and H-bond interactions. Arg254 contribute the most to the inhibitor selectivity among all residues. The energy difference of Arg254 in the A27S system is -1.22 kcal/mol, which is about 3.6 kcal/mol smaller than in other systems. The H-bond occupancy rate between Arg254 and the inhibitor is also 35% lower than others. In addition, Arg24 contributes to the inhibitor selectivity to a certain extent, as the energy difference in the A27S system is 1 kcal/mol smaller than other systems. Gln262 interacts with inhibitors via H-bonds in all system by basically equal energy contribution. For the K120A system, the energy differences of Tyr46, Asp181, and Phe182 are smaller than WT system, indicating that these residues will provide selectivity for the inhibitor. In addition, the H-bond occupancy rate between Phe182 and the inhibitor is also significantly different from other systems. These results indicate that the difference in Lys120 will affect the binding selectivity. However, this variation is compensated by the energy contributions of Ser216, which causes the selectivity provided by mutation K120A to be less than that of mutation A27S.

## 4. Conclusion 

Molecular dynamics simulations of A27S, K120A, and WT were performed to explore the key residue that affects the selectivity of bidentate inhibitors. Analysis of RMSF and clustering shows that Lys120 affects the flexibility of YRD-motif. By comparing the profile of distances between Lys120 and other residues in three systems, we found that Lys120 swings between YRD-motif and WPD-loop, acting as an aid to stabilize the orientation of side chains of Tyr46 and WPD-loop through hydrogen bonding with Tyr46 and Asp181, which in turn benefits the binding of the inhibitor. The absence of Lys120 results in a relatively unstable binding of the inhibitor at the active site. Results of binding free energy calculations and alanine scanning confirmed that the difference in binding affinity of the K120A system was mainly due to the contribution of Tyr46, Asp181, and Phe182. The A27S mutation at the second pTyr binding site affects the orientation Arg254 and Arg24 by forming H-bonds, which results in a conformational change in loop28-32. The distances between the inhibitor and the residues at the second pTyr binding site indicate that this effect ultimately leads to an unstable binding between the methyl salicylate of the inhibitor and Arg254. Alanine scanning and the H-bond analysis further determined that the different binding strength between Arg254 and the inhibitor is responsible for the variation of binding free energies. These results together demonstrate that the residue differences of Ala27/Ser29 and Lys120/Lys122 will affect the binding affinity of the inhibitor to some extent and can be applied in increasing the selectivity of the inhibitor to TCPTP.

## Figures and Tables

**Table 1:** Binding free energies (kcal/mol) and the individual energetic terms for the systems of WT, A27S, and K120A using the MM-PBSA method.

Energetic terms	WT	A27S	K120A
Δ*E*_vdW_	-47.55 ± 0.27	-43.26 ± 0.21	-46.12 ± 0.21
Δ*E*_ele_	-87.26 ± 0.19	-82.48 ± 0.54	-70.1 ± 0.22
Δ*G*_PB_	78.4 ± 0.23	75.38 ± 0.25	63.45 ± 0.19
Δ*G*_enpolar_	-33.23 ± 0.09	-31.73 ± 0.08	-32.61 ± 0.12
Δ*G*_edisper_	57.05 ± 0.08	55.07 ± 0.12	55.93 ± 0.09
^*a*^Δ*G*_solv_	102.22 ± 0.26	98.72 ± 0.29	86.77 ± 0.24
Δ*H*	-32.6 ± 0.22	-27.03 ± 0.25	-29.45 ± 0.27
-Δ*TS*	24.57 ± 0.39	22.16 ± 0.39	22.61 ± 0.4
Δ*G*_binding_	-8.03 ± 0.32	-4.86 ± 0.33	-6.84 ± 0.34
^*b*^Δ*G*_exp_	−7.33	−5.37 (TCPTP)	

^*a*^Δ*G*_solv_ = Δ*G*_PB_ + Δ*G*_enpolar_ + Δ*G*_edisper_. ^*c*^experimental binding free energy was calculated by Δ*G*_exp_ ≈ *RT *ln *K*_i_.

## Data Availability

The MD simulations data used to support the findings of this study are included within the article and the supplementary information file.

## Supplementary Materials

Supplementary Materials Fig. S1. Comparison of the correlation coefficient between Arg254 and other residues in the WT, A27S, and K120A system. Supplementary Fig. S2. Davies-Bouldin index (DBI) and the pseudo F-statistic (pSF) over the number of clusters for the simulation of WT, K120A, and A27S systems. Supplementary Fig. S3. Average structures of the top 10 clusters of (A)WT, (B)A27S, and (C)K120A systems superimposed at the active site, and average structures of the top 10 clusters of (D)WT, (E)A27S, and (F)K120A systems superimposed at loop28-32. Supplementary Fig. S4. Average structures of the WT, A27S, and K120A systems superimposed at the active site, shown in green, yellow, and blue, respectively. Supplementary Fig. S5. 2D protein-ligand interaction diagrams of the average structures for the WT (A), A27S (B), and K120A (C) systems. Supplementary Fig. S6. The distances between (A) the S*γ* atom of Cys215 and the O2 atom of the ligand, (B) the C*α* atom of Tyr46 and the C*α* atom of Asp181, (C) the O*η* atom of Tyr46 and the N*η*1 atom of Arg221, (D) N*η*2 atom of Arg254 and O6 atom of the ligand, and (E) N*ε*2 atom of Gln262 and O4 atom of the ligand. Supplementary Table S1. The occupancy of each cluster to the total conformations. Supplementary Table S2. Differences in binding free energies (kcal/mol) of WT, A27S, and K120A systems using the MM-PBSA alanine scanning method. Supplementary Table S3. Detailed information of H-bond between proteins and the ligand for WT, A27S, and K120A systems during the MD simulations.
