# Supplementary material for: Molecular Dynamics Simulations of A27S and K120A Mutated PTP1B Reveals Selective Binding of the Bidentate Inhibitor
Source: Biomed Res Int. 2019 Jan 8;2019:9852897. doi: 10.1155/2019/9852897 (PMC6341276; doi:10.1155/2019/9852897)
Supplement: Supplementary Materials — Fig. S1. Comparison of the correlation coefficient between Arg254 and other residues in the WT, A27S, and K120A system. Supplementary Fig. S2. Davies-Bouldin index (DBI) and the pseudo F-statistic (pSF) over the number of clusters for the simulation of WT, K120A, and A27S systems. Supplementary Fig. S3. Average structures of the top 10 clusters of (A)WT, (B)A27S, and (C)K120A systems superimposed at the active site, and average structures of the top 10 clusters of (D)WT, (E)A27S, and (F)K120A systems superimposed at loop28-32. Supplementary Fig. S4. Average structures of the WT, A27S, and K120A systems superimposed at the active site, shown in green, yellow, and blue, respectively. Supplementary Fig. S5. 2D protein-ligand interaction diagrams of the average structures for the WT (A), A27S (B), and K120A (C) systems. Supplementary Fig. S6. The distances between (A) the Sγ atom of Cys215 and the O2 atom of the ligand, (B) the Cα atom of Tyr46 and the Cα atom of Asp181, (C) the Oη atom of Tyr46 and the Nη1 atom of Arg221, (D) Nη2 atom of Arg254 and O6 atom of the ligand, and (E) Nε2 atom of Gln262 and O4 atom of the ligand. Supplementary Table S1. The occupancy of each cluster to the total conformations. Supplementary Table S2. Differences in binding free energies (kcal/mol) of WT, A27S, and K120A systems using the MM-PBSA alanine scanning method. Supplementary Table S3. Detailed information of H-bond between proteins and the ligand for WT, A27S, and K120A systems during the MD simulations. [file 9852897.f1.docx]

***Supporting information***

**Molecular Dynamics Simulations of A27S and K120A Mutated PTP1B Reveals Selective Binding Behavior of the Bidentate Inhibitor**

Xi Chen^a^, Qiang Gan^a^, Changgen Feng^a^, Xia Liu^b^, Qian Zhang ^a^

^a^ State Key Laboratory of Explosion Science and Technology, Beijing Institute of Technology, No.5, Zhongguancun South Street, Haidian District, Beijing 100081, China

^b^ College of Science, China Agricultural University, Beijing 100193, China

**Corresponding author**:

Qiang Gan, State Key Laboratory of Explosion Science and Technology, Beijing Institute of Technology, No.5, Zhongguancun South Street, Haidian District, Beijing 100081, China, e-mail: ganqiang@bit.edu.cn

Changgen Feng, State Key Laboratory of Explosion Science and Technology, Beijing Institute of Technology, No.5, Zhongguancun South Street, Haidian District, Beijing 100081, China, e-mail: cgfeng@cast.org.cn

**Contents:**

**Fig. S1.** Comparison of the correlation coefficient between Arg254 and other residues in the WT, A27S and K120A system. (*Page 1*)

**Table S1.** The occupancy of each cluster to the total conformations. (*Page 1*)

**Fig. S2.** Davies-Bouldin index (DBI) and the pseudo F-statistic (pSF) over the number of clusters for the simulation of WT, K120A, and A27S systems. (*Page 2*)

**Fig. S3.** Average structures of the top 10 clusters of (A)WT, (B)A27S and (C)K120A systems superimposed at the active site, and average structures of the top 10 clusters of (D)WT, (E)A27S and (F)K120A systems superimposed at loop28-32. (*Page 3*)

**Fig. S4.** Average structures of the WT, A27S, and K120A systems superimposed at the active site, which shown in green, yellow and blue, respectively. (*Page 3*)

**Fig. S5.** 2D protein-ligand interaction diagrams of the average structures for the WT **(A)**, A27S **(B)** and K120A **(C)** systems. (*Page 4*)

**Fig. S6.** The distances between **(A)** the Sγ atom of Cys215 and the O2 atom of the ligand, **(B)** the Cα atom of Tyr46 and the Cα atom of Asp181, **(C)** the Oη atom of Tyr46 and the Nη1 atom of Arg221, **(D)** Nη2 atom of Arg254 and O6 atom of the ligand, and **(E)** Nε2 atom of Gln262 and O4 atom of the ligand. (*Page 5*)

**Table S2.** Differences in binding free energies (kcal/mol) of WT, A27S and K120A systems using the MM-PBSA alanine scanning method. (*Page 6*)

**Table S3.** Detailed information of H-bond between proteins and the ligand for WT, A27S and K120A systems during the MD simulations. (*Page 6*)





**Fig. S1.** Comparison of the correlation coefficient between Arg254 and other residues in the WT, A27S and K120A system.

**Table S1.** The occupancy of each cluster to the total conformations.

| Cluster No. | Occupancy (%) | | |
| --- | --- | --- | --- |
|  | WT | A27S | K120A |
| 1 | 13.24 | 13.5 | 10.52 |
| 2 | 10.48 | 8.13 | 9.12 |
| 3 | 9.14 | 8.82 | 8.61 |
| 4 | 8.21 | 6.34 | 7.26 |
| 5 | 5.89 | 6.34 | 7.01 |
| 6 | 5.45 | 6.3 | 6.97 |
| 7 | 5.4 | 6.24 | 6.33 |
| 8 | 4.72 | 6.22 | 5.03 |
| 9 | 4.46 | 5.62 | 4.72 |
| 10 | 3.8 | 5.55 | 4.53 |
| Total | 70.79 | 73.06 | 70.1 |





**Fig. S2.** Davies-Bouldin index (DBI) and the pseudo F-statistic (pSF) over the number of clusters for the simulation of WT, K120A, and A27S systems.


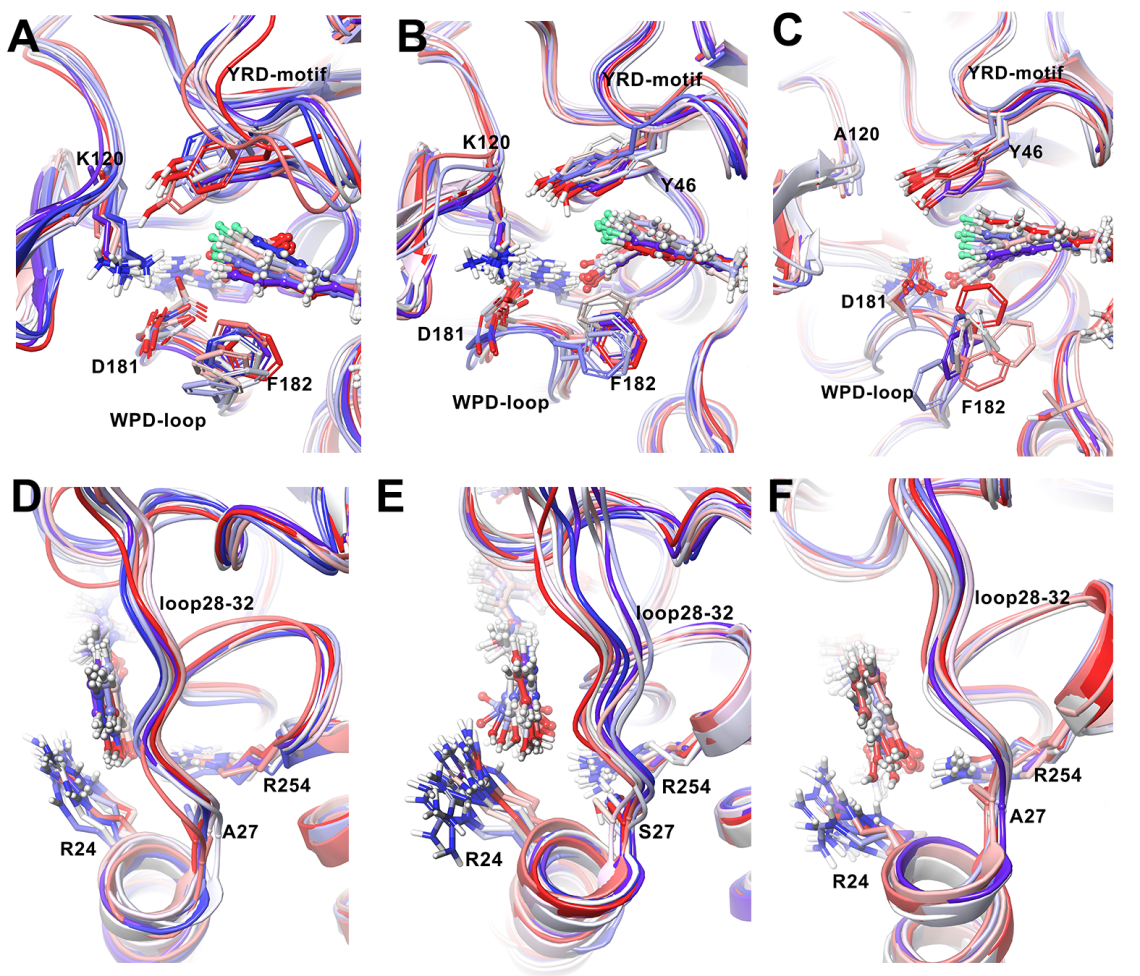


**Fig. S3.** Average structures of the top 10 clusters of (A)WT, (B)A27S and (C)K120A systems superimposed at the active site, and average structures of the top 10 clusters of (D)WT, (E)A27S and (F)K120A systems superimposed at loop28-32. The structure is colored from red to blue, representing the cluster from large to small.


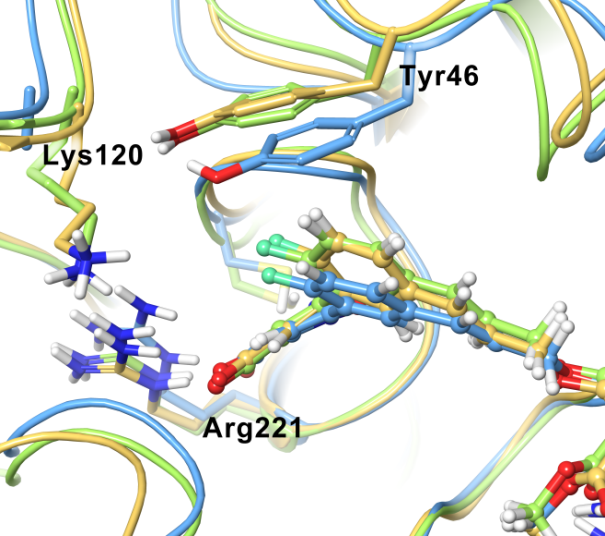


**Fig. S4.** Average structures of the WT, A27S, and K120A systems superimposed at the active site, which shown in green, yellow and blue, respectively.


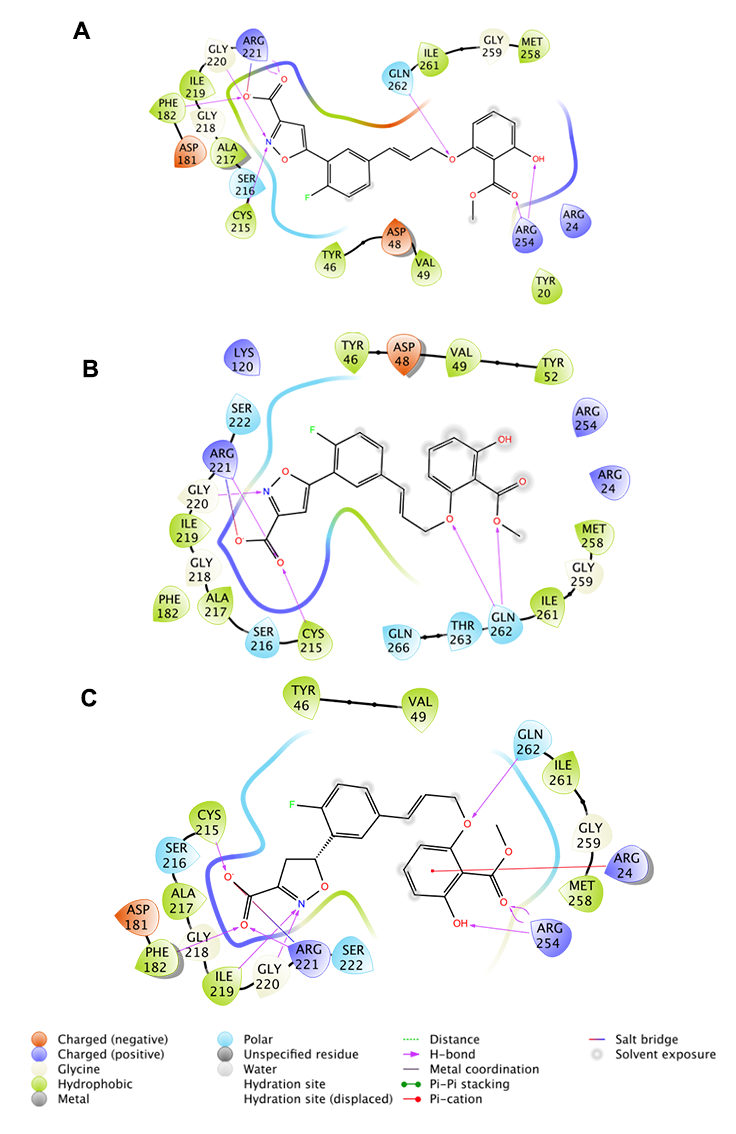


**Fig. S5.** 2D protein-ligand interaction diagrams of the average structures for the WT **(A)**, A27S **(B)** and K120A **(C)** systems. The interactions were analyzed by Maestro, Schrödinger (LLC, New York, NY, 2017).

**
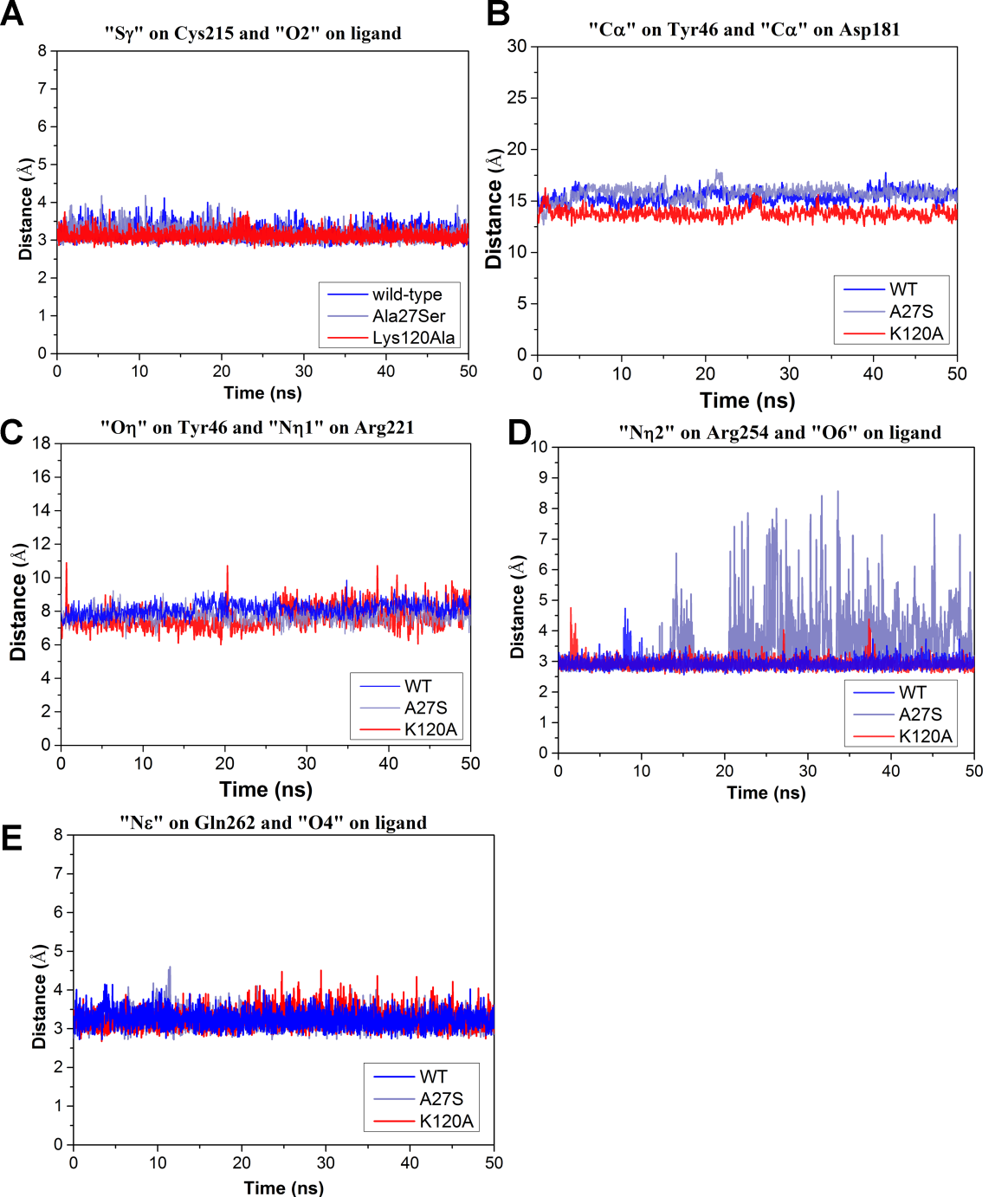
**

**Fig. S6.** The distances between **(A)** the Sγ atom of Cys215 and the O2 atom of the ligand, **(B)** the Cα atom of Tyr46 and the Cα atom of Asp181, **(C)** the Oη atom of Tyr46 and the Nη1 atom of Arg221, **(D)** Nη2 atom of Arg254 and O6 atom of the ligand, and **(E)** Nε2 atom of Gln262 and O4 atom of the ligand.

**Table S2.** Differences in binding free energies (kcal/mol) of WT, A27S and K120A systems using the MM-PBSA alanine scanning method.

| **Residue** | **WT** | **A27S** | **K120A** |
| --- | --- | --- | --- |
| R24A | -3.52 ± 0.34 | -2.45 ± 0.18 | -3.56 ± 0.10 |
| Y46A | -2.15 ± 0.12 | -2.46 ± 0.12 | -0.96 ± 0.21 |
| K120A | -0.88 ± 0.001 | -0.62 ±0.001 | -- |
| D181A | 2.04 ± 0.20 | 2.01 ± 0.20 | 3.5 ± 0.30 |
| F182A | -4.16 ± 0.24 | -4.04 ± 0.18 | -3.23 ± 0.22 |
| C215A | -4.8 ± 0.16 | -4.45 ± 0.17 | -4.1 ± 0.13 |
| S216A | -1.43 ± 0.25 | -2.43 ± 0.25 | -2.97 ± 0.20 |
| R221A | -24.95 ± 0.27 | -24.39 ± 0.28 | -25.53 ± 0.47 |
| R254A | -4.88 ± 0.22 | -1.22 ± 0.22 | -5.24 ± 0.20 |
| Q262A | -5.11 ± 0.23 | -5.61 ± 0.23 | -5.56 ± 0.26 |

**Table S3.** Detailed information of H-bond between proteins and the ligand for WT, A27S and K120A systems during the MD simulations.

| **System** | **Acceptor** | **Donor** | **Fraction (%)** | **AvgDist** | **AvgAng** |
| --- | --- | --- | --- | --- | --- |
| **WT** | ligand O1 | Arg221 N1 | 93.58 | 2.7608 | 155.8537 |
|  | ligand O6 | Arg254 N2 | 71.46 | 2.8513 | 153.6922 |
|  | ligand O2 | Arg221 N | 54.07 | 2.8677 | 166.3541 |
|  | ligand N1 | Gly220 N | 48.96 | 2.9135 | 152.1057 |
|  | ligand O1 | Arg221 Nε | 38.72 | 2.8733 | 146.6261 |
|  | ligand O7 | Arg254 N1 | 19.8 | 2.8991 | 144.7992 |
|  | ligand O2 | Arg221 Nε | 19.22 | 2.8958 | 163.8559 |
|  | ligand O1 | Ser216 Og | 10.16 | 2.7242 | 163.8909 |
|  | ligand O6 | Arg254 N1 | 17.2 | 2.8979 | 146.1774 |
|  | ligand O5 | Gln262 N2 | 14.26 | 2.9196 | 151.6404 |
|  | ligand O7 | Arg24 N | 6.92 | 2.9183 | 151.8804 |
| **A27S** | ligand O1 | Arg221 N1 | 85.22 | 2.7933 | 150.0945 |
|  | ligand O2 | Arg221 N | 74.79 | 2.8537 | 165.6499 |
|  | ligand O1 | Arg221 N | 62.46 | 2.8444 | 146.5629 |
|  | ligand N1 | Gly220 N | 38.08 | 2.9262 | 150.0610 |
|  | ligand O6 | Arg254 N2 | 35.73 | 2.8460 | 153.8120 |
|  | ligand O2 | Arg221 N | 34.97 | 2.8990 | 156.9943 |
|  | ligand O4 | Gln262 N2 | 12.71 | 2.9216 | 152.6159 |
|  | ligand O7 | Arg254 N1 | 8.7 | 2.9062 | 147.2624 |
|  | ligand O6 | Arg254 N1 | 4.62 | 2.9004 | 145.7132 |
|  | ligand O1 | Phe182 N | 4.12 | 2.8909 | 160.0673 |
|  | ligand O5 | Gln262 N2 | 2.59 | 2.9216 | 145.3192 |
|  | ligand O7 | Arg24 N | 1.35 | 2.9172 | 149.9206 |
| **K120A** | ligand O2 | Arg221 N | 88.96 | 2.8348 | 167.1229 |
|  | ligand O1 | Arg221 N | 75.42 | 2.8126 | 148.7846 |
|  | ligand O6 | Arg254 N2 | 70.77 | 2.8479 | 152.8184 |
|  | ligand O1 | Phe182 N | 62.11 | 2.8524 | 163.1413 |
|  | ligand O1 | Arg221 N1 | 45.75 | 2.8435 | 145.5599 |
|  | ligand N1 | Gly220 N | 36.40 | 2.9260 | 148.0831 |
|  | ligand O6 | Arg254 N1 | 22.48 | 2.8964 | 146.2966 |
|  | ligand O2 | Arg221 N | 19.36 | 2.9129 | 156.2725 |
|  | ligand O7 | Arg254 N1 | 18.02 | 2.8956 | 144.1281 |
|  | ligand O4 | Gln262 N2 | 11.07 | 2.9229 | 150.3608 |
|  | ligand O7 | Arg24 N | 2.87 | 2.9104 | 150.8599 |
